# Clinical study of duloxetine hydrochloride combined with doxazosin for the treatment of pain disorder in chronic prostatitis/chronic pelvic pain syndrome: An observational study

**DOI:** 10.1097/MD.0000000000006243

**Published:** 2017-03-10

**Authors:** Mingxin Zhang, Hanzhong Li, Zhigang Ji, Dexin Dong, Su Yan

**Affiliations:** Department of Urology, Chinese Academy of Medical Sciences, Peking Union Medical College Hospital, Beijing, China.

**Keywords:** management, men, psychological problems, symptoms, variables

## Abstract

To explore the safety and efficacy of the selective 5-serotonin and norepinephrine reuptake inhibitor duloxetine hydrochloride and alpha-adrenergic receptor blocker (alpha-blocker) doxazosin mesylate-controlled tablets in the treatment of pain disorder in chronic prostatitis/chronic pelvic pain syndrome (CP/CPPS).

In all, 150 patients were enrolled and 126 patients completed the study (41 patients in the doxazosin group, 41 patients in the sertraline group, and 44 patients in the duloxetine group). This was an open randomized 6-month study. CP/CPPS patients who met the diagnostic criteria were randomized into 3 groups. The patients in the duloxetine group received doxazosin 4 mg + duloxetine 30 mg once a day, and the dosage of duloxetine was increased to 60 mg after a week. The patients in the doxazosin group received doxazosin 4 mg once a day. The patients in the sertraline group received doxazosin 4 mg + sertraline 50 mg once a day. National Institutes of Health Chronic Prostatitis Symptom Index (NIH-CPSI) score, the short-form McGill Pain questionnaire (SF-MPQ), and the hospital anxiety and depression scale (HAD) were applied for evaluations during follow-up of 1, 3, and 6 months after treatment.There were slight positive significant correlations between NIH-CPSI scores and HAD scores, moderate positive significant correlations between the quality of life (QOL) and SF-MPQ, and slight positive significant correlations between HAD and QOL. The effective rate in the doxazosin group was 4.88%, 19.51%, and 56.10% after 1, 3, and 6 months, respectively (*P* < 0.05). The SF-MPQ score in the doxazosin group decreased to 1.80 ± 1.29, 2.66 ± 1.57, and 3.24 ± 1.67 after 1, 3, and 6 months, respectively (*P* < 0.05). The HAD score in the doxazosin group decreased to 2.24 ± 2.17, 4 ± 2.11, and 4.90 ± 2.62 after 1, 3, and 6 months, respectively (*P* < 0.05). The effective rate in the sertraline group was 9.76%, 36.59%, and 63.41% after 1, 3, and 6 months, respectively. The SF-MPQ score in the sertraline group decreased to 1.76 ± 1.28, 3.07 ± 2, and 3.93 ± 2.53 after 1, 3, and 6 months, respectively (*P* < 0.05). The HAD score in the sertraline group decreased to 3.56 ± 4.11, 5.73 ± 5.26, and 7.27 ± 6.50 after 1, 3, and 6 months, respectively (*P* < 0.05). The effective rate in the duloxetine group was 36.36%, 88.64%, and 88.64% after 1, 3, and 6 months, respectively. The SF-MPQ score in the duloxetine group decreased to 3.61 ± 2.54, 6.05 ± 3.66, and 7.41 ± 4.26 after 1, 3, and 6 months, respectively (*P* < 0.05). The HAD score in the duloxetine group decreased to 3.14 ± 3.28, 6.93 ± 3.90, and 9.43 ± 4.67 after 1, 3, and 6 months, respectively (*P* < 0.05). There were significant differences in the reduction of the NIH-CPSI score and the SF-MPQ score between the duloxetine group and the sertraline group and between the duloxetine group and the doxazosin group (*P* < 0.01). There were significant differences in the reduction of the HAD score at 3 months between the duloxetine group and the doxazosin group, and there were significant differences in the reduction of the HAD score at 6 months among the groups (*P* < 0.05). The incidence rates of adverse reactions in the duloxetine group, the sertraline group, and the duloxetine group were 29.5%, 17%, and 7.3%, respectively, with adverse events ranging from mild to moderate.

There was a clear relationship between the extent of pain and mental factors in CP/CPPS with the main symptom of pain. Doxazosin combined with duloxetine exhibited good safety and efficacy in the treatment of pain disorder in CP/CPPS.

## 1 Introduction

More than 90% of symptomatic patients have chronic prostatitis/chronic pelvic pain syndrome (CP/CPPS) with the main symptom being pain in the pelvic region.^[1]^ The pain is persistent or recurrent and is located in the lower abdomen, perineum, testicles, or penis. With the change of seasons or course of disease, the extent and characteristics of the pain can fluctuate or change.^[2]^ The symptoms that last more than 3 months in the past 6 months, with or without various voiding symptoms and sexual dysfunction.^[3]^ Many treatment methods, such as antibiotics, alpha-blockers, and nonsteroidal anti-inflammatory drugs, have been used widely in the treatment of CPPS but showed low efficiency; particularly in the treatment of the pain of CPPS, the efficiency was limited and temporary.^[4]^ The physical discomfort and great economic burden caused by recurrent and incurable diseases cause serious damage to the patient's quality of life (QOL).

Since the tricyclic antidepressants were found to have antidepressant properties, many antidepressant drugs have been used in the treatment of chronic pain.^[5]^ Antidepressant drugs directly or indirectly have effects on opioid, histamine, cholinergic, 5-serotonin, and other receptors or ion channels to exert their analgesic effect.^[6]^ Several randomized, double-blind, placebo-controlled studies have confirmed that tricyclic antidepressants such as amitriptyline have good analgesic effects. Duloxetine hydrochloride is a new selective 5-serotonin and norepinephrine reuptake inhibitor (SNRI) antidepressant drug with good efficacy and tolerability in treating a variety of types of chronic pain and is a recommended first-line drug by the International Association for the Study of Pain for chronic pain syndromes such as diabetic peripheral neuropathic pain, postherpetic neuralgia, and fibromyalgia.^[7]^ Duloxetine hydrochloride combined with the alpha-blocker doxazosin was used for the treatment of chronic pain in CPPS, and the control groups were a doxazosin group and a sertraline (the selective 5-serotonin reuptake inhibitor [SSRI] sertraline hydrochloride combined with doxazosin) group to explore its safety and efficacy.

## 2 Materials and methods

### 2.1 Experimental design

This was a prospective, open label study with 3 phases. Phase I is a week-long screening and enrollment period. Phase II is the treatment period, which will last for 6 months. The eligible patients will be randomly divided into 3 equal-sized groups through a voice telephone system aided by computer. The groups are a doxazosin group (doxazosin 4 mg qd), a doxazosin + sertraline group (doxazosin dosage is the same as in the doxazosin group, and sertraline is 50 mg/d; according to the efficacy and tolerability, the dosage can be adjusted to 25 mg or 100 mg), and a doxazosin + duloxetine group (doxazosin dosage is the same as in the doxazosin group, and duloxetine is 30 mg/d; the dosage is increased to 60 mg/d after a week; then, the dosage can be adjusted to 120 mg/d, if the treatment is invalid or if the patient cannot tolerate the dosage, the dosage can be adjusted back to 60 mg/d). Phase III is the withdrawal period, which lasts for 2 weeks. The patients who completed the treatment will move into this period. The purpose of the withdrawal period is to reduce the incidence of withdrawal reactions, and the dosage will be reduced gradually until the withdrawal is complete. The patients in the 3 groups will be followed up at 1, 3, and 6 months to assess efficacy and safety.

The experimental design was developed according to the moral principles and implementation of the Declaration of Helsinki. The Peking Union Medical College Hospital Ethics Committee requires informed consent for the experiment, and there are no experiments performed before receiving signed informed consent. Informed consent was obtained from all individual participants included in the study.

### 2.2 Inclusion criteria

Those male patients aged above 18 years and diagnosed with CP/CPPS in the Department of Urology in our hospital with discomfort or pain symptoms in the pelvic area for at least 3 months will be enrolled. The National Institutes of Health Chronic Prostatitis Symptom Index (NIH-CPSI) score should be ≥15 points,^[8]^ and NIH-CPSI pain score should be ≥4 points.^[9]^

The main exclusion criteria are as follows: treatment with doxazosin or other alpha-adrenergic receptor blockers for CP/CPPS or for any other reason in the past; urinary tract infection (>100,000 CFU bacteria/mL in a urine culture); genital herpes in the past 3 months; 5α-reductase inhibitor for 3 months in the past year; unilateral testicular pain without pelvic area symptoms; urinary or reproductive system cancer; inflammatory bowel disease; active urethral stricture; prostate or bladder operation history; neurogenic bladder; or the usage of a strong P-3A4 enzyme inhibitor (ketoconazole, itraconazole, ritonavir, etc.) or erythromycin. Those patients with mania, bipolar disorder, psychosis, or who were identified as a suicide risk by researchers were excluded. In addition, those patients who used monoamine oxidase inhibitors or who had liver disease or other serious diseases in the 2 weeks before screening were also excluded.

### 2.3 Groups

The subjects were enrolled from January 2011 to January 2012, and the last follow-up time was July 2012. A total of 150 patients were enrolled under the criteria, and all patients were randomly divided in to 3 treatment groups, the doxazosin group, the sertraline group, and the duloxetine group; there were 50 patients in each group. A total of 24 patients did not complete the experiment (9 in the doxazosin group, 9 in the sertraline group, and 6 in the duloxetine group), and the main reasons were adverse drug reactions in 8 patients (33.3%, 2 patients in the doxazosin group, 3 patients in the sertraline group, and 3 patients in the duloxetine group); poor efficacy in 9 patients (37.5%, 5 patients in the doxazosin group, 3 patients in the sertraline group, and 1 patient in the duloxetine group); and loss to follow-up in 7 patients (29.2%, 3 in the doxazosin group, 2 in the sertraline group, and 2 in the duloxetine group).

### 2.4 Evaluation criteria

The main index was the NIH-CPSI score. The curative index required that the NIH-CPSI score improved by ≥ 25% and decreased by at least 6 points, as recommended by Nickel et al.^[10]^ The total possible score on the NIH-CPSI scale is 43 points, and it takes into account 3 major symptoms: pain (location, frequency, and severity; 0–21), urination (irritation and obstructive symptoms; 0–10), and adverse effect on QOL (0–12).^[8]^ In the short-form McGill pain questionnaire (SF-MPQ), the total scores are 0 to 45 and include sensory 0 to 33 and affective 0 to 12 descriptors; the higher the score, the more severe the pain.^[11]^ The hospital anxiety and depression scale (HAD) was also utilized (the total scores are 0–42, the higher the score, the more severe the anxiety and depression).^[12]^

### 2.5 Safety assessment

Adverse events are briefly reviewed according to the toxicity standard and organ system. The toxicity effects of each subject and each system are evaluated, and each system of each participant is evaluated as 1 adverse event. If a particular participant has multiple adverse events in the same organ, the most serious events will be recorded. The adverse events in each experimental group were evaluated.

### 2.6 Statistical analysis

IBM SPSS Statistics software is utilized for statistical analysis. Single factor analysis of variance is used for the index comparison among the 3 groups, and the assumption of homogeneous variance for the LSD method is used for multiple comparisons. T tests of paired sample indexes are used for the comparison of indexes are different time points. Bivariate correlation analysis is used for the analysis of SF-MPQ, NIH-CPSI, and HAD. Pearson analysis is used to calculate the correlation coefficient. A 2-sided significance test is used; *P* < 0.01 is considered highly significantly different, and *P* < 0.05 is considered significantly different.

## 3 Results

### 3.1 Baseline of the patients in each group

The baseline data of the patients in the 3 groups are shown in Table 1. The average ages of the patients in the doxazosin group, sertraline group, and duloxetine group are 33.59, 32.51, and 32.78 years old, respectively, with no significant difference. There were no significant differences in the mean NIH-CPSI baseline score among groups, and there were no significant differences in the HAD or SF-MPQ baseline score among groups.

**Table 1:**
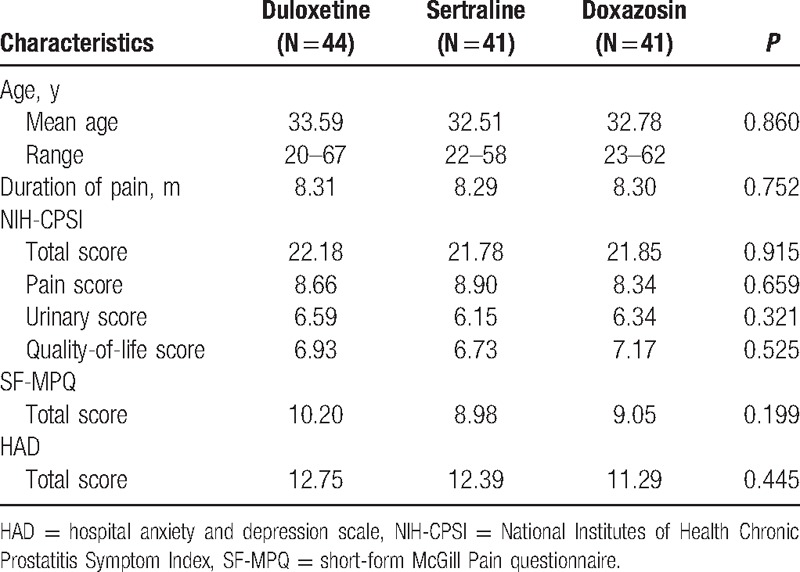
Baseline characteristics of the subjects in the 3 groups.

### 3.2 Correlation between the total NIH-CPSI and HAD scores and the SF-MPQ and HAD QOL scores

**Figure 1:**
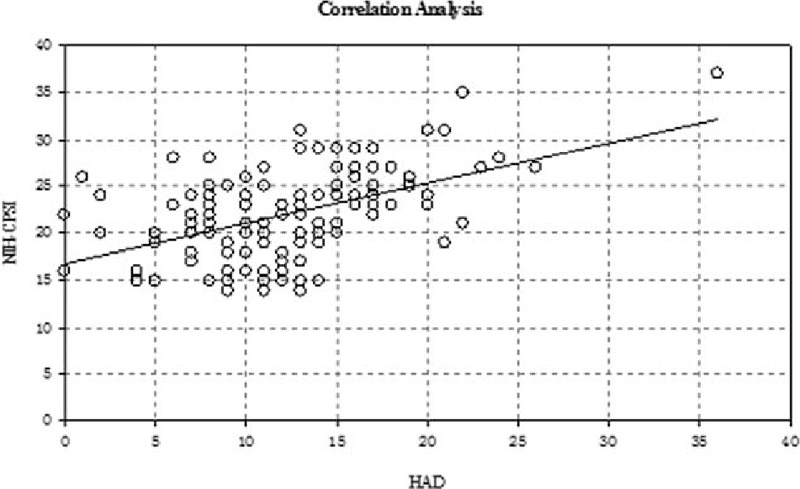
Correlation analysis between NIH-CPSI score and HAD score. HAD = hospital anxiety and depression scale, NIH-CPSI = National Institutes of Health Chronic Prostatitis Symptom Index.

**Figure 2:**
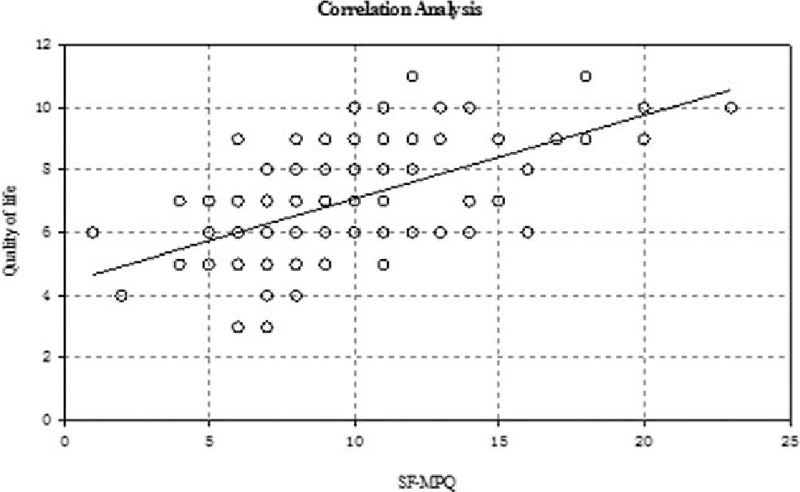
Correlation analysis between the quality of life of NIH-CPSI and SF-MPQ. NIH-CPSI = National Institutes of Health Chronic Prostatitis Symptom Index, SF-MPQ = short-form McGill Pain questionnaire.

**Figure 3:**
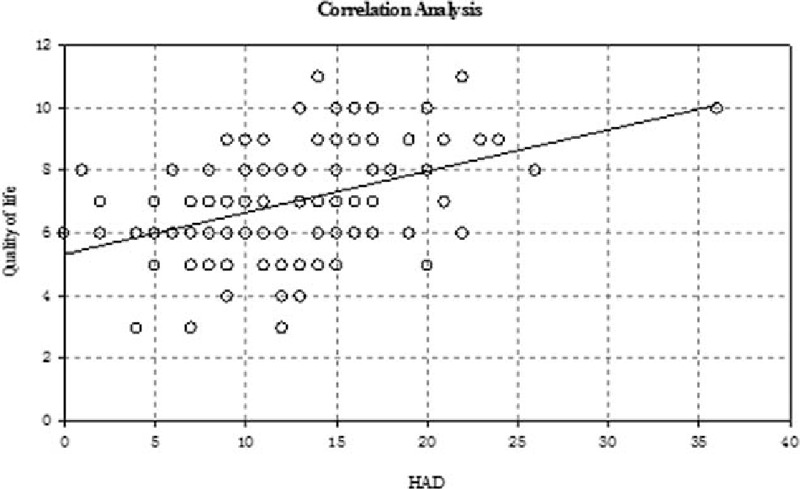
Correlation analysis between HAD score and quality of life index. HAD = hospital anxiety and depression scale.

**Figure 4:**
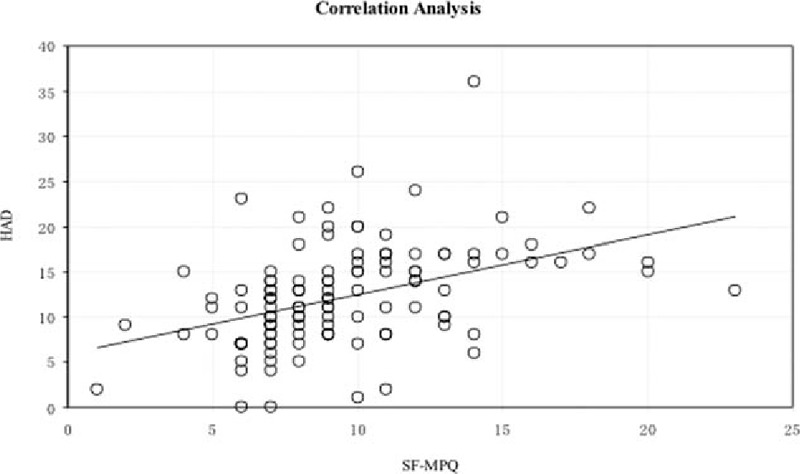
Correlation analysis between HAD score and SF-MPQ. HAD = hospital anxiety and depression scale, SF-MPQ = short-form McGill Pain questionnaire.

There was a slight positive and significant correlation between the total NIH-CPSI score and the HAD score, the HAD score increased with the increase of the NIH-CPSI score (Fig. 1). There was a moderate and significant correlation between QOL and SF-MPQ (Fig. 2). Furthermore, there was a slight positive correlation between HAD and QOL (Fig. 3), and there was a slight positive correlation between SF-MPQ and HAD (Fig. 4).

### 3.3 Changes in the NIH-CPSI total score

The effective rates of doxazosin at 1, 3, and 6 months after treatment were 4.88%, 19.51%, and 56.10%, respectively; the effective rates of sertraline were 9.76%, 36.59%, and 63.41%, respectively; and the effective rates of duloxetine were 36.36%, 88.64%, and 88.64%, respectively. The effective rates and changes of NIH-CPSI are shown in Table 2.

**Table 2:**
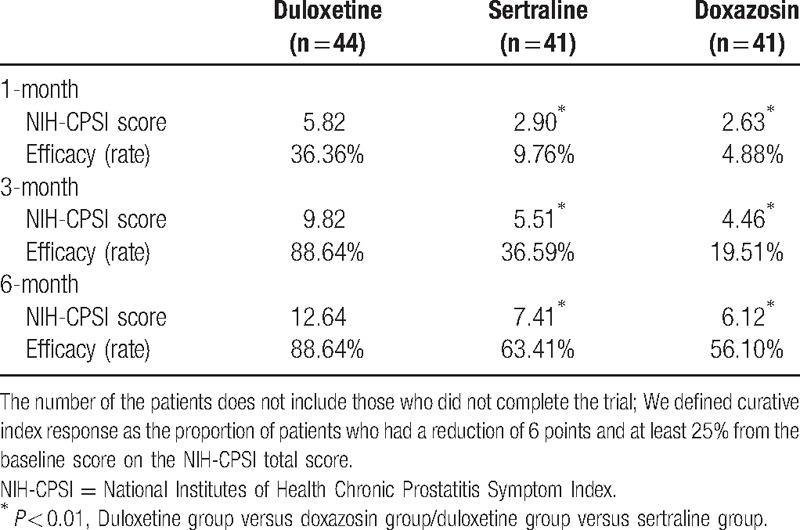
Reduction of NIH-CPSI score and the efficacy rate for 126 patients.

The NIH-CPSI scores decreased gradually with prolonged treatment, and the duloxetine group had the largest reduction and the highest efficiency at each time point; the doxazosin group had the smallest reduction, and the reduction in the sertraline group was intermediate. There were significant differences between the duloxetine group and sertraline group and between the duloxetine group and doxazosin group at each time point (*P* < 0.01), but there was no significant difference between the sertraline group and doxazosin group (*P* = 0.602, *P* = 0.135, and *P* = 0.145).

### 3.4 Changes in SF-MPQ

The McGill Pain scores improved significantly 1, 3, and 6 months after treatment compared with those before treatment in the doxazosin, sertraline, and duloxetine groups (*P* < 0.01) (see changes in Table 3).

**Table 3:**
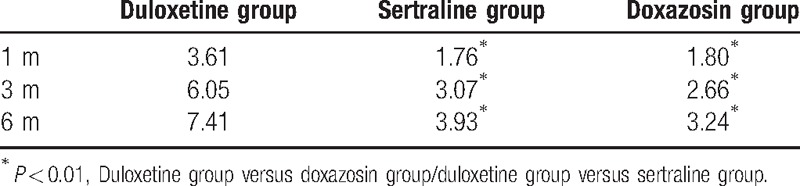
Reduction of the McGill Pain Questionnaire score.

There were significant differences in the decline of McGill Pain scores between duloxetine group and sertraline group and between the duloxetine group and the doxazosin group at each time points in 1, 3, and 6 months (*P* < 0.01), while there was no significant difference between the sertraline group and the doxazosin group (*P* = 0.904, *P* = 0.473, and *P* = 0.314).

### 3.5 Changes in HAD scores

There was significant improvement between groups at 1, 3, and 6 months compared with the baseline after treatment (*P* < 0.01). There were no significant reductions in HAD score for any group at 1 month. After 3 months, There were no significant differences between the duloxetine group and sertraline group (*P* = 0.166) or between the sertraline group and the doxazosin group (*P* = 0.051), but the HAD score improved significantly in the duloxetine group compared with the doxazosin group (*P* < 0.01). The HAD score improved significantly in the duloxetine group compared with the sertraline group (*P* = 0.042), in the sertraline group compared with the doxazosin group (*P* = 0.029), and in the duloxetine group compared with doxazosin group after 6 months (*P* < 0.01). The changes in HAD scores are shown in Table 4.

**Table 4:**
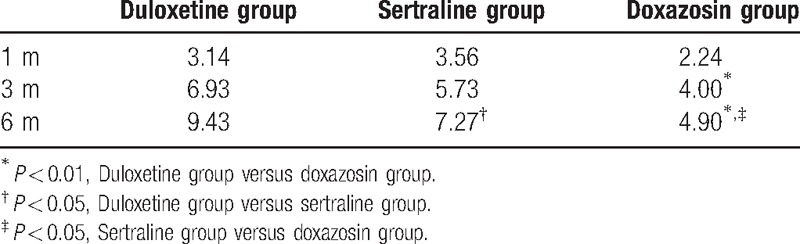
Reduction of the hospital anxiety and depression scores.

### 3.6 Safety evaluation

The common adverse reactions included dizziness, nausea, dry mouth, constipation, lethargy, vomiting, and palpitations. There were no serious adverse reactions; most adverse reactions were mild to moderate, and most weakened gradually until they disappeared with prolonged administration. The majority of the patients could tolerate 3 to 5 days after oral administration, but 8 patients dropped out of the trial because of adverse drug reactions (3 in the duloxetine group, 3 in the sertraline group, and 2 in the doxazosin group). The occurrence rate of nausea and vomiting was 15.91% in duloxetine group, which was significantly higher than that in the other 2 groups, and some patients were unable to tolerate the adverse reactions. Details of common types and frequencies of adverse effects are shown in Table 5.

**Table 5:**
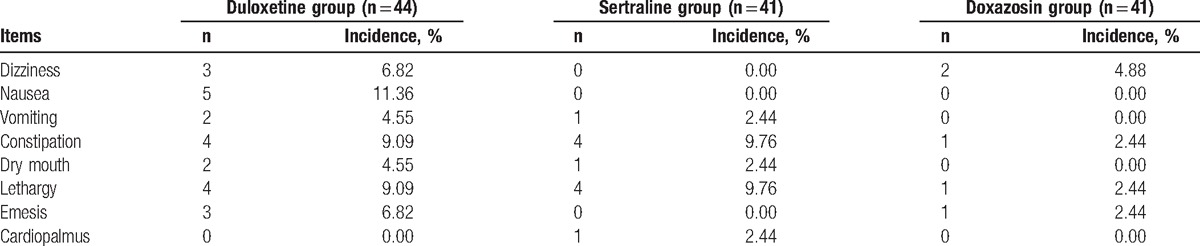
Rates of adverse events.

## 4 Discussion

In our study, a prospective and randomized trial was designed to explore efficacy and safety by comparing the SNRI drug duloxetine hydrochloride and the SSRI drug sertraline hydrochloride combined with doxazosin in the treatment of pain disorder in CP/CPPS, and the results were also compared with the commonly used alpha-blocker doxazosin mesylate in CP/CPPS. Giannantoni et al^[13]^ studied 38 patients with CP/CPPS by randomized controlled methods and used NIH-CPSI, International Index of Erectile Function-5 (IIEF-5) questionnaires, the Hamilton Anxiety Scale (HAM-A), and the Hamilton Depression Scale (HAM-D) as the evaluation tools. Patients in group 1 received tamsulosin (0.4 mg/d), saw palmetto (320 mg/d), and duloxetine (60 mg/d), and the patients in group 2 received tamsulosin (0.4 mg/d) and saw palmetto (320 mg/d). The NIH-CPSI score and subscores improved significantly compared with baseline after 16 weeks, while the patients in group 2 only showed significant improvement in NIH-CPSI and urinary evaluation score. Significant improvements in the NIH-CPSI pain subscore, the NIH-CPSI QOL subscore, and the NIH-CPSI total score were observed in patients in group 1 compared with those in group 2 (*P* < 0.01), together with a significant improvement in HAM-A and HAM-D scores (*P* < 0.01). By contrast, our study had larger sample size, longer observation time and used the McGill Pain score as an additional pain assessment tool.

The deterioration of psychoemotional status frequently accompanies urologic symptoms and pain in patients with CP/CPPS; chronic pain and depressive symptoms in patients with CP/CPPS, alone or in combination, are associated with a risk of diminished physical functioning and deterioration of QOL.^[14,15]^ Our study showed that there was a slight positive and significant correlation between the NIH-CPSI score and the HAD score and between the HAD and SF-MPQ. There was a significant positive correlation between the HAD and QOL, which indicated that the extent of pain in CPPS with pain as the main symptom was closely associated with mental factors, and mental factors are emerging as the most important factors affecting the QOL.

In recent years, alpha-blockers have been widely used in the treatment of CP/CPPS and are regarded as a basic drug for CPPS. The mechanism of alpha-blockers may lie in the following 2 aspects: Firstly, the continuous and frequent contraction of the bladder neck and prostate smooth muscle, regulated mainly by alpha-adrenergic receptors, may be one of the mechanisms of CP/CPPS, and alpha-blockers can relax the prostate, urethra, and bladder smooth muscle tissue. Secondly, the excitement of alpha-adrenergic receptors in the central nervous system can delay pain relief in CP/CPPS, and alpha-blockers can reduce the neuroinflammation in the lower urinary tract.^[16]^ Li et al^[17]^ studied the efficacy of 4 alpha-blockers, including prazosin hydrochloride, terazosin hydrochloride, phenoxybenzamine hydrochloride, and doxazosin mesylate in the treatment of CP/CPPS using a randomized controlled study in which the control group did not receive an alpha-blocker, and the curative effect was statistically significant. Cheah et al^[18]^ observed 86 CP/CPPS patients treated with terazosin for 14 weeks using a random, placebo-controlled method, and terazosin significantly improved the NIH-CPSI score, pain score, urinary symptoms, and QOL score. A large randomized, multicenter, double-blind, placebo-controlled study selected 272 CP/CPPS patients who had never had alpha-blockers before. The patients received alfuzosin treatment for 12 weeks, but alfuzosin did not show obvious advantages compared with placebo in the NIH-CPSI score and its 3 subscores.^[19]^ Based on the effective index being a reduction of more than 6 points, the present study showed that the NIH-CPSI score did not improve significantly 1 and 3 months after doxazosin treatment, but it improved significantly after 6 months. SF-MPQ and HAD scores improved to a certain degree without significant differences. This result indicated that alpha-blockers could relieve the symptoms of CP/CPPS to some degree, but the therapeutic effect was not significant. Further treatment methods should be explored.

The mechanism of antidepressant drugs on chronic pain is direct action on neurons for pain and indirect improvement of depression, anxiety, and other emotional disorders, thereby improving the experience of pain and the ability to cope with pain. An epidemiological survey of chronic pain has clearly suggested that depressive symptoms and pain experience are closely related, especially in chronic pelvic pain. Some scholars believe that nervous people, with physiological and genetic origins of anxiety, are easier to diagnose with the presence of anxiety, depression, and other affective disorders induced by chronic pain.^[20]^ In a randomized, placebo-controlled study, 14 male patients with chronic pelvic pain received 26 weeks of treatment with sertraline 50 mg daily. The results showed some improvement in symptoms but were not significantly different.^[21]^ Our study indicated that there were no significant differences in NIH-CPS scores or SF-MPQ between the sertraline combined with doxazosin group and the doxazosin group at 1, 3, and 6 months, and there was no significant difference in HAD scores at 1 and 3 months, but there was significant difference at 6 months.

Our study indicated that there was significant improvement in the NIH-CPS score, SF-MPQ, and HAD after treatment with the new antidepressant duloxetine hydrochloride combined with doxazosin after 1, 3, and 6 months. In addition, the improvement was also significant compared with doxazosin and sertraline group. There is now evidence that 5-hydroxytryptamine (5-HT) and norepinephrine (NE) regulate pain through endogenous descending pain inhibitory pathways, and their dysfunction is a potential mechanism in patients experiencing pain. Antidepressant drugs are used for many chronic pain syndromes through adding 5-HT- and NE-mediated neurotransmission. Previous studies have shown that the double effect of inhibition of 5-HT and NE mediated by antidepressants is clearly superior to single receptor therapy. Duloxetine hydrochloride is a powerful 5-HT and NE reuptake inhibitor with a relatively balanced affinity for 5-HT and NE, and animal experiments have shown that duloxetine lacks an obvious affinity for cholinergic, adrenergic, and opiate receptors, and its analgesic effects on neuropathic pain are better than those of venlafaxine, amitriptyline, and other antidepressants.^[22,23]^ 5-HT and NE nerve conduction abnormalities play an important role in the pathogenesis of severe depression, and they often coexist with CP. Serious depression in patients often presents with painful physical symptoms, such as headache, backache, and chronic pelvic pain, and duloxetine can markedly relieve the pain of severe depression with somatic symptoms.^[24]^

The dosage, course of treatment, and possible adverse reactions were communicated to the patients and adjusted before treatment, and a few patients chose other treatment methods because of adverse reactions after this communication. The majority of patients in the sertraline group had mild and tolerable adverse reactions, such as dizziness, nausea, vomiting, and constipation after 2 to 3 days, which disappeared gradually in 5 to 7 days. In the duloxetine group, there was nausea, vomiting, constipation, and lethargy, and we used the so-called “sandwich” method, that is, administration during a meal significantly reduced the incidence of gastrointestinal reaction. Most adverse events were transient and mild, weakened gradually, and disappeared in approximately 1 week.

Antidepressant drugs are mainly used for mental disease, and they are still in the exploratory stage for the treatment of CP. We should strictly control the indication of treatment, obtain the informed consent of patients and strictly follow-up to facilitate timely adjustment of the treatment scheme. Literature reviews^[20,25]^ and the present study recommend that the indications for the use of antidepressants in the treatment of pain disorders in CPPS are as follows: First, the disease is associated with obvious anxiety, depression, and other psychological symptoms, and cognitive behavior therapy is invalid. Second, the main symptom is persistent or recurrent pain, and the use of alpha-blockers and galenical drugs was invalid, seriously affecting the QOL of the patients.

This study also has limitations. First, the samples were small in the 3 groups, which may have had adverse effects on the determination of results. Second, a placebo group was not established because of patient compliance and ethical constraints in 6 months in real clinical practice, which might have a certain bias on the interpretation and safety assessment. Previous studies have shown that the efficiency of placebo in CP/CPPS is up to 30%,^[19]^ and the effective rate in the sertraline and duloxetine groups in this study was >30%, which confirmed the effectiveness of the treatment.

Duloxetine has been widely used in chronic pain, especially in neuropathic pain, but reports are rare for the treatment of CP/CPPS. With respect to chronic pain, especially for neuropathic pain, the therapeutic efficacy for 1 type of pain is not exactly the same as for another type of pain. However, the efficacy of first-line drugs that have been confirmed in 1 or more types of pain may be reasonable for another type of pain, and the drugs that are needed in clinical practice should exhibit satisfactory efficacy.^[7]^ CP/CPPS is caused by a variety of pathogenic factors, and a single treatment method is unlikely to be satisfactory; a variety of drugs and/or the combined application of various methods is rational.^[26]^ The SNRI drug duloxetine hydrochloride combined with the alpha-blocker doxazosin was safe and effective in the treatment of pain disorder in CP/CPPS, which is useful information in the treatment of CP/CPPS, and more exact curative effects need to be confirmed by more rigorous clinical studies.
